# Supplementary material for: MetaRibo-Seq measures translation in microbiomes
Source: Nat Commun. 2020 Jun 29;11:3268. doi: 10.1038/s41467-020-17081-z (PMC7324362; doi:10.1038/s41467-020-17081-z)
Supplement: Supplementary file 10 — Supplementary Data 7 [file 41467_2020_17081_MOESM10_ESM.zip › File2/Confidence_High_Taxonomy/97406_out.krona.html]

Javascript must be enabled to view this page.

members
magnitude
magnitudeUnassigned
count
unassigned
taxon
rank

97406\_out

1

1
superkingdom
2759

33208
1
kingdom

1
phylum
7711

subphylum
1
89593

8287
superclass
1

40674
class
1

314146
superorder
1

order
1
9989

1
suborder
1963758

family
1
10066

1
subfamily
39107

10088
genus
1

862507
subgenus
1

1
species

SRS077552\_contig\_number\_23105
10090
